# Targeting the Wnt signaling pathway through R-spondin 3 identifies an anti-fibrosis treatment strategy for multiple organs

**DOI:** 10.1371/journal.pone.0229445

**Published:** 2020-03-11

**Authors:** Mingjun Zhang, Michael Haughey, Nai-Yu Wang, Kate Blease, Ann M. Kapoun, Suzana Couto, Igor Belka, Timothy Hoey, Matthew Groza, James Hartke, Brydon Bennett, Jennifer Cain, Austin Gurney, Brent Benish, Paola Castiglioni, Clifton Drew, Jean Lachowicz, Leon Carayannopoulos, Steven D. Nathan, Jorg Distler, David A. Brenner, Kandasamy Hariharan, Ho Cho, Weilin Xie

**Affiliations:** 1 Celgene Corporation, San Diego, CA, United States of America; 2 OncoMed Pharmaceuticals, Redwood City, CA, United States of America; 3 Celgene Corporation, Summit, NJ, United States of America; 4 Advanced Lung Disease and Transplant Program, Inova Fairfax Hospital, Falls Church, VA, United States of America; 5 Department of Internal Medicine, University of Erlangen-Nuremberg, Erlangen, Germany; 6 Department of Medicine, University of California San Diego, La Jolla, CA, United States of America; National Institutes of Health, UNITED STATES

## Abstract

The Wnt/β-catenin signaling pathway has been implicated in human proliferative diseases such as cancer and fibrosis. The functions of β-catenin and several other components of this pathway have been investigated in fibrosis. However, the potential role of R-spondin proteins (RSPOs), enhancers of the Wnt/β-catenin signaling, has not been described. A specific interventional strategy targeting this pathway for fibrosis remains to be defined. We developed monoclonal antibodies against members of the RSPO family (RSPO1, 2, and 3) and probed their potential function in fibrosis *in vivo*. We demonstrated that RSPO3 plays a critical role in the development of fibrosis in multiple organs. Specifically, an anti-RSPO3 antibody, OMP-131R10, when dosed therapeutically, attenuated fibrosis in carbon tetrachloride (CCl_4_)-induced liver fibrosis, bleomycin-induced pulmonary and skin fibrosis models. Mechanistically, we showed that RSPO3 induces multiple pro-fibrotic chemokines and cytokines in Kupffer cells and hepatocytes. We found that the anti-fibrotic activity of OMP-131R10 is associated with its inhibition of β-catenin activation *in vivo*. Finally, RSPO3 was found to be highly elevated in the active lesions of fibrotic tissues in mouse models of fibrosis and in patients with idiopathic pulmonary fibrosis (IPF) and nonalcoholic steatohepatitis (NASH). Together these data provide an anti-fibrotic strategy for targeting the Wnt/β-catenin pathway through RSPO3 blockade and support that OMP-131R10 could be an important therapeutic agent for fibrosis.

## Introduction

Fibroproliferative diseases (fibrosis) can occur in almost any organ and are one of the major causes of patient morbidity and mortality [1]. The approval of pirfenidone and nintedanib for IPF, currently the only approved treatments for any fibrotic diseases, represented a significant advancement in the treatment paradigm for IPF. However, the clinical benefits of these treatments seem to be limited to delaying progression in only a subset of patients. Superior therapies for IPF and other chronic diseases characterized by excessive tissue fibrosis are urgently needed [2, 3].

Wnt/β-catenin signaling has been shown to play an important role in mouse models of fibrosis of lung, liver, kidney, and skin, and it has been implicated in the development of fibrosis in humans [4–12]. Genetic deletion of components in the Wnt signaling complexes inhibits fibrosis [8, 12], and overexpression of certain Wnt ligands or β-catenin in epithelial cells or fibroblasts induces fibrosis in animals [5, 6]. Therefore, modulating Wnt signaling represents an attractive anti-fibrosis strategy. However, the complexity of a signaling network consisting of 19 Wnt ligands, 10 FZD receptors, and many secreted antagonists and agonists as well as its homeostatic role in certain normal tissues, has presented a major challenge in the development of therapies targeting this pathway for fibrosis or any other disease.

R-spondins (RSPO 1–4) are the secreted proteins that belong to R-spondin protein family and are enhancers of Wnt signaling [13]. RSPOs bind to the leucine-rich repeat-containing G protein-coupled receptors, LGR 4, 5, 6. [14] and stabilize the Wnt receptors and co-receptors by inactivation of the membrane-bound E3 ubiquitin ligases RNF43 and ZNRF3, which target the Wnt receptors for ubiquitination-mediated degradation [15,16]. All four RSPO proteins can activate canonical Wnt signaling through similar mechanism. As the role of Wnt signaling pathway in fibrosis has been well documented, we sought to investigate if any of the RSPO proteins play any role. Previously RSPO1 and 2 were reported to be up-regulated in a CCl_4_-induced liver fibrosis model [17, 18], however, the biological consequence of RSPO1 and RSPO2 expression has not been described. There has been no direct examination of their potential function in fibrosis. The studies described here probed the *in vivo* role of RSPO proteins in fibrosis using anti-RSPO selective monoclonal antibodies. We report that RSPO3 plays an important role in the development of fibrosis in multiple organs and provide evidence supporting that a RSPO3 functional blocking antibody could be a therapeutic agent to treat fibrosis.

## Materials and methods

### Anti-RSPO antibodies

The generation and characterization of anti-RSPO1, 2, and 3 were previously described [19]. Briefly, purified human RSPO1, 2, and 3 proteins were used to immunize mice, followed by hybridoma generation and characterization.

### Human tissue specimens

Human idiopathic pulmonary fibrosis (IPF) lung tissue samples were obtained from Dr. Steven D. Nathan’s laboratory at Inova Fairfax Hospital (Falls Church, VA) from studies that were approved by the Inova Fairfax Hospital Institutional Review Board. Human normal lung tissue samples were purchased from Avaden BioSciences (Scarsdale, NY) and Proteogenex (Culver City, CA). Human normal and NASH liver tissue samples were obtained from Dr. Anna Mae Diehl’s laboratory at Duke University School of Medicine (Durham, NC). All of the normal and patient tissue samples were reviewed by pathologists for approvals.

### Fibrosis models

Bleomycin-induced skin fibrosis model. Six-week old male DBA/2 mice from Janvier (Le Genest-Saint-Isle, France), were maintained in specific pathogen-free conditions with sterile pellet food, water, and a normal day-night cycle. Skin fibrosis was induced by daily subcutaneous injections of bleomycin (2.5 mg/kg) for three weeks. To evaluate the effects of nintedanib on established fibrosis, robust fibrosis was first induced by injections of bleomycin for three weeks. Thereafter, treatment was initiated, while injections of bleomycin were continued. All test antibodies including control antibodies were given in three doses of 25 mg/kg i.p. per week. Treatment with nintedanib, applied in doses of 50 mg/kg p.o. bid, was served as positive control. The outcome was analyzed six weeks after the first injection of bleomycin. Mice injected with 0.9% NaCl, the vehicle of bleomycin, were served as non-fibrotic controls.

For prophylactic dosing of anti-RSPO1, 2 and 3, liver fibrosis was induced in male C57B1/6J mice via intraperitoneal (IP) injection of CCl_4_ (0.75 mL/kg) in mineral oil (15% v/v CCl_4_ in mineral oil). Carbon tetrachloride (CCl_4_) was injected 3 times a week and always at least 4 hours after IP dosing of mineral oil or test articles. Mice received 25 mg/kg of anti-RSPO1, anti-RSPO2, OMP-131R10, or isotype control once a week in a prophylactic mode starting from Day 0. The naïve (control/mineral oil) group did not undergo injection of CCl_4_ but received 0.75 mL/kg of mineral oil via IP injection once a week. Mice were sacrificed using CO_2_ at Day 28. After sacrifice, the liver lobes were collected and isolated. The medial liver lobes were then snap frozen and stored at -80°C.

For the therapeutic dosing of OMP-131R10, liver fibrosis was induced in male C57B1/6J mice via intraperitoneal (IP) injection of CCl_4_ (0.75 mL/kg) in mineral oil (15% v/v CCl_4_ in mineral oil). Isotype control was diluted in vehicle 1 consisting of 40 mM histidine, 130 mM sucrose, 150 mM sodium, and 0.02% Tween. The naïve (control/mineral oil) groups did not undergo injection of CCl_4_. Additional groups that were tested in this study included an aqueous carboxymethyl cellulose (CMC)/Tween control group, and pirfenidone and lysyl oxidase-like 2 (LOXL2) antibody were used as reference compounds. After sacrifice, blood was collected by cardiac puncture and samples were stored at -80°C until shipment to Celgene Corporation, San Diego, CA. Liver lobes (caudate, lateral, and medial right liver lobes) were collected and isolated prior to snap freezing and stored at -80°C until shipment to Celgene Corporation.

Bleomycin-induced lung fibrosis. In order to induce pulmonary fibrosis, mice were administered 2 U/kg of clinical grade bleomycin (blenoxane, catalog number NDC 0703-3154-01 TEVA Pharmaceutical Works Ltd (Hungary) via oropharyngeal route. Animals from control group were administered 70 μL of saline oropharyngeally, and they were served as sham controls. Bleomycin (15U) from a sealed bottle was resuspended in 1 mL of PBS. A fresh stock solution of 15 U/mL was used and diluted in saline to prepare a dose of 1.5U/kg in a 70 μL volume for each mouse. Briefly, mice were anesthetized with isoflurane inhalant anesthesia. Seventy μL of bleomycin was then administered into the back of the oral cavity with a syringe using a blunt needle. The animal was then returned to its cage and monitored until fully recovered from the anesthesia.

The studies were performed by TNO (Amsterdam, Netherlands) with the approval of TNO IACUC committee. Euthanasia was performed using CO2 asphyxiation followed by exanguination via cardiac puncture.

### Liver histology and collagen content assay

The left lateral liver lobe was fixed for 48 hours in 4% formaldehyde and embedded in paraffin. Five μm sections were stained using Picro Sirius Red. Two complete sections were scanned using a Leica Aperio AT2 scanner. The surface area of Sirius Red positive collagen was determined using Image J software (rsbweb.nif.gov/ij) version 1.48v) with a custom made macro. Collagen and protein content were determined in the medial left liver lobes. After acid hydrolysis, hydroxyproline and protein were determined using chromogenic assays (Quickzyme Biosciences. Leiden, The Netherlands; Total Collagen Assay) according to the manufacturer’s instructions.

### Evaluation of dermal thickness

After the animals were sacrificed by cervical dislocation, skin samples of 1 cm^2^ were obtained from a defined area of the upper back between shoulder blades. Lesional skin areas were excised, fixed in 4% formalin for 6 h, and embedded in paraffin. Five μm sections were cut and stained with hematoxylin and eosin. The dermal thickness was measured at 100-fold magnification by measuring the distance between the epidermal-dermal junction and the dermal-subcutaneous fat junction at three sites from lesional skin of each mouse.

### Detection of myofibroblasts

Myofibroblasts are characterized by the expression of α-smooth muscle actin (α-SMA). α-SMA positive fibroblasts were detected by incubation with monoclonal anti-α-SMA antibodies (clone 1A4, Sigma-Aldrich, Steinheim, Germany). The expression was visualized with horseradish peroxidase labeled secondary antibodies and 3,3-diaminobenzidine tetrahydrochloride (DAB) (Sigma-Aldrich). Monoclonal mouse IgG antibodies (Calbiochem, San Diego, CA, USA) were used for controls.

### Hydroxyproline assay

The amount of collagen protein in skin samples was determined via hydroxyproline assay. After digestion of punch biopsies (Ø 3 mm) in 6 M HCl for three hours at 120°C, the pH of the samples was adjusted to 6 with 6 M NaOH. Afterwards, 0.06 M chloramine T was added to each sample and incubated for 20 min at room temperature. Next, 3.15 M perchloric acid and 20% p-dimethylaminobenzaldehyde were added and samples were incubated for additional 20 min at 60°C. The absorbance was determined at 557 nm with a Spectra MAX 190 microplate spectrophotometer.

### BAL leukocyte differentiation

BAL leukoyctes were evaluated using Differential Quik Stain (Electron Microscopy Sciences) according to the manufacturer’s protocol. Differential counts were performed using a microscope (20x objective) with approximately 100 cells counted (macrophage, lymphocyte, eosinophil and neutrophil). Percent cellular composition calculated and populations calculated using this percent from the total cells retrieved.

### Gene expression analysis

Human bronchial epithelial cells (Science Cell Research, San Diego, CA) or human pulmonary alveolar epithelial cells (Science Cell Research, San Diego, CA) were cultured in T-150 flasks in BEpiCM growth medium and allowed to reach 80% confluency. Cells were plated in 12-well plastic culture plates at 150,000 cells per well in BEpiCM medium for 24 hours. OMP-131R10 was formulated at a final pH of 6.0 in 40 mM histidine/130 mM sucrose/150 mM sodium chloride (NaCl)/0.02% Tween. Cells were then stimulated with 100 ng/mL recombinant Wnt3a (formulated in phosphate buffered saline [PBS]), 350 pM RSPO3 (formulated in PBS) or a combination of Wnt3a and RSPO3 for 24 hours. Ribonucleic acid (RNA) was isolated using a Qiagen Rneasy Mini Kit according to manufacturer’s instruction. Axin2 and MMP-7 gene expression was determined using reverse transcription polymerase chain reaction (RT-PCR) Taq-Man assays. Quantitative PCR (qPCR) was performed using SuperScript^®^ III One-Step RT-PCR System and ran on a Viia 7 Real-Time PCR System. Data was normalized to glyceraldehyde 3-phosphate dehydrogenase.

### Cytokine secretion assays

Primary mouse hepatocytes (Cell Biologics, Chicago, IL) and mouse Kupffer cells (EMD Millipore, Burlington, MA) were treated with 10 ng/ml mouse R-Spondin 3 (R&D Systems, Minneapolis, MN) for 48 hours. Supernatants were collected, and cytokines were measured using mouse Proinflammatory Panel 1 and mouse Cytokine Panel 1 (Mesoscale, Rockville, MD).

### Tissue staining

DAB single color immunohistochemistry (IHC) staining of RSPO1-3 (Atlas Antibody. Rabbit anti-RSPO1, HPA046154, 1:50; rabbit anti-RSPO2, HPA024764, 1:50; rabbit anti-RSPO3, HPA029957, 1:250), α-SMA (mouse anti-α-SMA, Sigma, A5228, 1:15K), and β-catenin (rabbit anti-β-catenin, Abcam, ab32572, 1:500), with normal rabbit (Abam, ab37415 & ab172730) and mouse (Abcam, ab18413) IgG as isotype controls, were performed on the Bond-Max Autostainer (Leica Microsystems) with Bond polymer refine detection kit (Leica, DS9800). Formalin-fixed, paraffin-embedded (FFPE) tissue sections (4 μm) were antigen-unmasked with epitope retrieval solution 1 (Leica, AR9961) for RSPO1-3 and α-SMA, or epitope retrieval solution 2 (Leica, AR9961) for β-catenin, for 20 minutes at 100°C, and blocked with peroxide blocking agent for 5 minutes. Sections were incubated with primary antibodies at appropriate dilutions for 15 minutes. For α-SMA staining on mouse tissues, a secondary rabbit anti mouse IgG antibody (Abcam, ab133469, 1:500) was applied for 8 minutes. Sections were then incubated in polymer for 8 minutes, developed in DAB for 10 minutes, counterstained with hematoxylin for 5 minutes, and then coverslipped with Sakura Finetek. Multiplex immunofluorescent (IF) staining of RSPO1/RSPO2/RSPO3/α-SMA and RSPO3/CD45/F4.80 were performed on Leica Bond-Rx Autostainer (Leica Microsystems) with Opal 7-Color Automation IHC Kit (PerkinElmer, NEL821001KT). FFPE tissue sections (4 μm) were antigen-unmasked with epitope retrieval solution 1 for 20 minutes at 100°C, blocked with PKI blocking buffer for 5 minutes. Anti-RSPO3 antibody was applied for 30 minutes at room temperature, followed by Opal Polymer HRP for 10 minutes, and Opal 690 reagent for 10 minutes. The tissue sections were then heated again in epitope retrieval solution 1 for 20 minutes at 95°C to remove excessive unbounded antibody before they were stained for the next antibody. For RSPO1/RSPO2/RSPO3/α-SMA co-IF, the similar staining cycle was repeated for RSPO2 with Opal 620, RSPO1 with Opal 540, and α-SMA with Opal 570 sequentially. For α-SMA staining on mouse tissues, a secondary rabbit anti mouse IgG antibody was applied for 10 minutes. For RSPO3/CD45/F4.80 co-IF, the similar staining cycle was repeated for CD45 (eBioscience, 14-0451-82, 1:125) with Opal 540, and F4.80 (eBioscience, 14-4801-81, 1:500) with Opal 620 sequentially. For CD45 and F4.80 antibodies, a secondary rabbit anti rat IgG antibody (Vector Lab, AI-4001, 1:200) was applied for 10 minutes. Finally the slides were coverslipped with Prolong gold antifade reagent with DAPI (Invitrogen, P36935), and images were taken with Vectra Polaris (PerkinElmer). PicroSirius Red staining was performed on 4 μm FFPE sections with PSR stain kit (American Mastertech, KTPSRPT). Two complete sections were scanned using a 3DHistech Pannoramic Midi Scanner. The surface area of Sirius Red positive collagen was determined using ImageJ software (rsbweb.nih.gov/ij/; version 1.48v) with a custom made macro.

### Statistical analysis

Correlational analysis of RSPO3 Immunohistochemistry scores and percentage of forced vital capacity was performed using Pearson's product-moment correlation coefficient. P values were calculated using the unpaired Student’s t test for normally distributed data. Graphs were created using GraphPad Prism 6.0. For the *in vivo* studies with fibrosis models, a one-way ANOVA followed by a Dunnett’s multiple comparisons test comparing groups to the isotype control group was used.

## Results

### RSPO3 is highly expressed in active lesions of fibrotic livers

**Fig 1:**
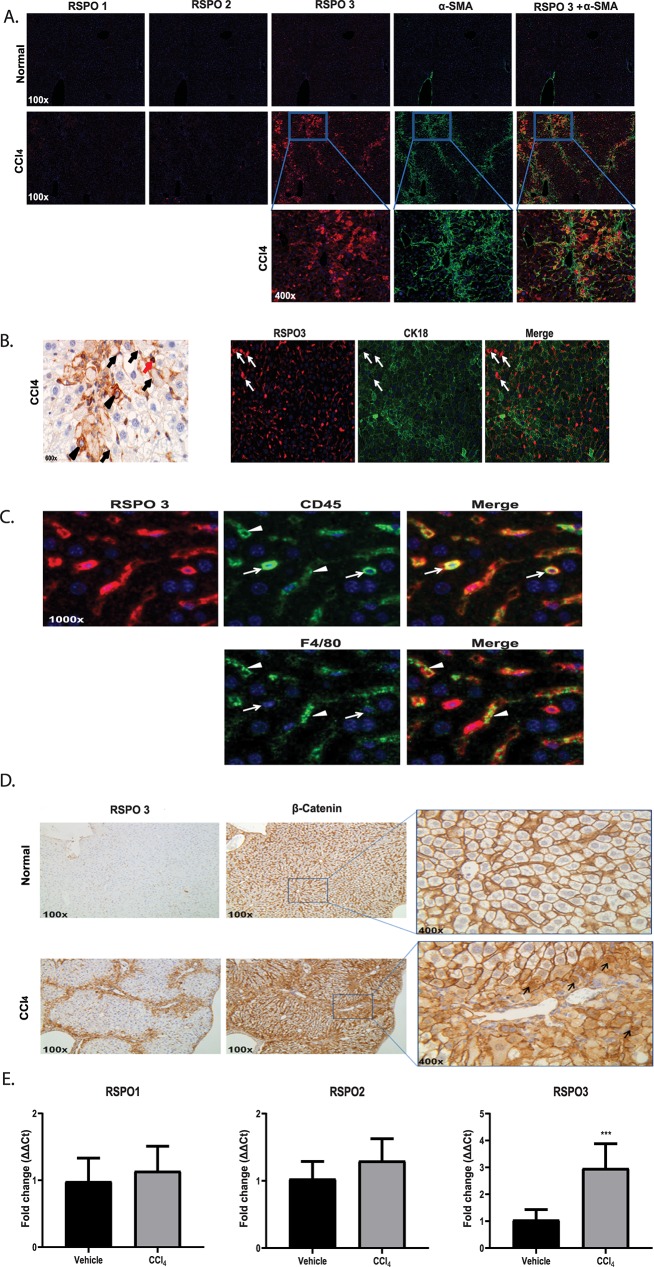
R-Spondin 3 is highly expressed in mouse fibrotic livers. **A**, Representative fluorescent immunohistochemistry of RSPO1, RSPO2, RSPO3 and α-SMA expression in the livers of mice treated with CCl_4_ or vehicle control. **B,** RSPO3 expression pattern in the livers of mice treated with CCl_4_. Hepatic stellate cells (black arrow), inflammatory cells (red arrow), damaged hepatocytes (black arrowhead). Co-immunofluorescent staining of RSPO3 and CK18 was shown in damaged hepatocytes (white arrow). **C,** Expression of RSPO3 on CD45+ or F4/80 cells in CCl_4_ mouse livers. **D,** β-catenin expression pattern in the livers of mice treated with CCl_4_. β-catenin nuclear translocation (black arrow). **E,** RT-PCR of RSPO1, RSPO2 and RSPO3. Values are presented as means ± Stdev. n = 8–10 mice per group. Statistical analysis was conducted using unpaired t- test. ****P <0.0001.

To investigate a potential role for RSPO proteins in fibrosis, we began by examining the expression of RSPO 1, 2 and 3 in CCl_4_-induced liver fibrosis by immunohistochemistry (IHC). All three antibodies and staining conditions were validated and optimized (S1–S6 Figs). In normal mouse livers, little or non-detectable signals were observed for all three RSPO proteins (Fig 1A). RSPO3 was reported to express in the central vein area of normal liver using RNA in situ hybridization [20]. We believe the discrepancy on RSPO3 expression in normal liver likely comes from the sensitivity of different detection methods. However in livers from animals administrated with CCl_4_, RSPO3 was found to be markedly increased while minimum changes were seen for RSPO1 or RSPO2 (Fig 1A). Overlay of staining for RSPO3 and α-smooth muscle actin (α-SMA), a marker of activated fibroblast or hepatic stellate cell, showed that highly expressed RSPO3 largely resides within fibrotic lesions (Fig 1A). In addition, hepatocytes and inflammatory cells, including macrophages, were also found to express RSPO3 (Fig 1B and 1C). Upregulation of β-catenin and increased cytoplasmic and nuclear translocation were found in livers from CCl_4_-treated mice, particularly in areas where RSPO3 was overexpressed, suggesting RSPO3 overexpression may play a role in the activation of the canonical Wnt signaling pathway (Fig 1D). While the β-catenin cytoplasmic and nuclear translocation was clearly observed in hepatocytes in CCl_4_-treated mouse livers, little or no such effect was found in mesenchymal cells such as hepatic stellate cells. We believe this may largely be due to the IHC detection limitation under current experimental condition for these cells that are small in size. The induction of RSPO3, but not RSPO1 or RSPO2, in CCl_4_ livers was further confirmed by qPCR analysis of mRNAs (Fig 1E). These findings led us to test the potential function of RSPO proteins, in particular RSPO3, in fibrosis *in vivo*.

### RSPO3 plays an important role in liver fibrosis through activation of the Wnt/β-catenin signaling pathway

Because of the severe phenotypes from RSPO gene knockout studies including embryonic lethality from RSPO3 gene disruption, we generated and used selective monoclonal antibodies against each of three RSPO proteins (RSPO1-3) to test their effects in animal models of fibrosis.

**Fig 2:**
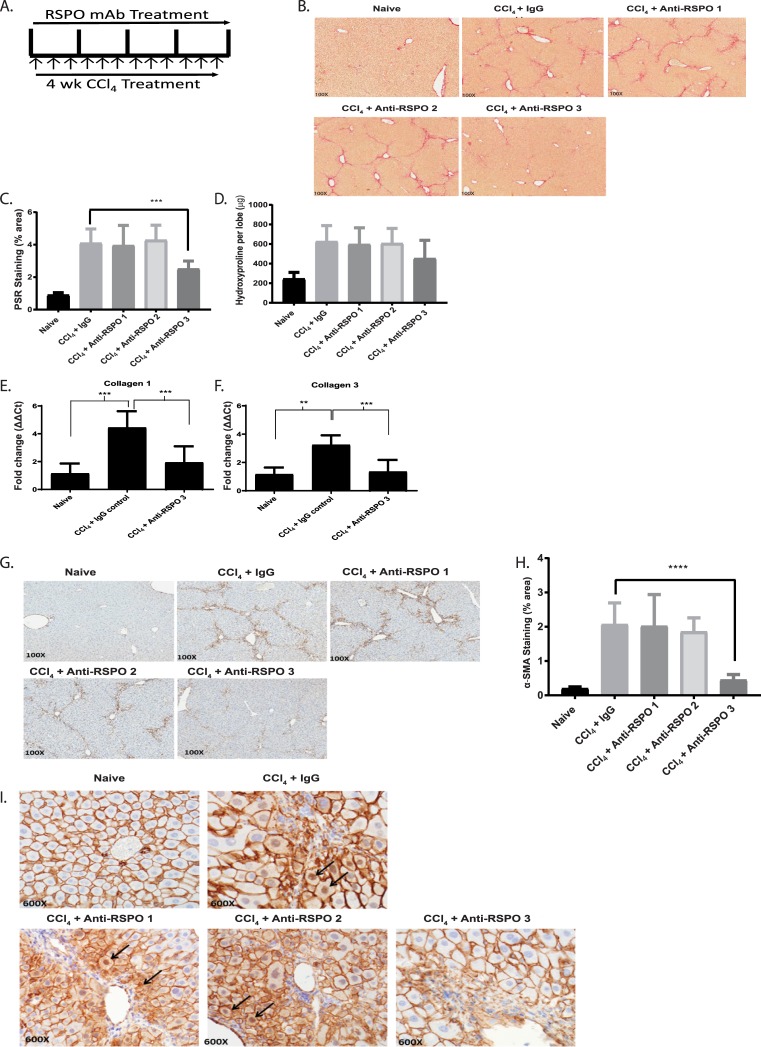
RSPO3 plays an important role in liver fibrosis through activation of the Wnt/β-catenin signaling pathway. **A,** Treatment scheme of anti-RSPO antibodies in CCl_4_ liver fibrosis model. **B,** Representative Picro Sirius Red staining of normal and CCl_4_ induced mouse livers treated with anti-RSPO1, anti-RSPO2, and OMP-131R10. **C,** Quantification of Picro Sirius Red staining area. **D**, Quantification of liver collagen content. **E,** RT-PCR of collagen I. **F,** RT-PCR of collagen III. **G,** Representative immunohistochemistry analysis of α-SMA in the livers of normal and CCl_4_ induced mouse livers treated with anti-RSPO1, anti-RSPO2 and OMP-131R10. **H,** Quantification of α-SMA staining area. **I,** Immunohistochemistry analysis of β-catenin. Nuclear β-catenin were detected in hepatocytes (arrow) in active fibrotic areas. Liver fibrosis was induced by CCl_4_ injection three times weekly for 4 weeks The mice were treated from day 1 with anti-RSPO1, anti RSPO2 or anti- RSPO3 (OMP-131R10) antibodies at 25 mg/kg once a week (n = 10 animals per group except mineral oil control n = 7). At week 4, the study was terminated and liver levels of hydroxyproline were measured by biochemical methods and percent Picro Sirius Red (PSR), and then quantitated via histological methods. Data is shown as mean ± SEM and % inhibition calculated when compared to the vehicle group following subtraction of the mineral oil control. Statistical comparison was done using one-way ANOVA with Dunnett’s multiple comparisons test using the Isotype as a control. *** = p < 0.001; **** = p < 0.0001.

The specificity and potency of each anti-RSPO antibodies were determined by their inhibition of Wnt target gene expression in mouse epithelial cells induced by combination of each RSPO protein and Wnt3a. All three antibodies were shown to be specific to their corresponding mouse RSPO proteins (S7 Fig). We next tested anti-RSPO1, 2, and 3 antibodies *in vivo* in a CCl_4_-induced mouse model of liver fibrosis with prophylactic dosing [21]. Animals were treated with 25 mg/kg, once a week (QW) for over four weeks, of anti-RSPO antibodies at the same time as the initiation of liver injury by CCl_4_ administration (Fig 2A). We found that anti-RSPO3 monoclonal antibody, OMP-131R10, but not antibodies against RSPO1 or 2, significantly protected the animals from developing liver fibrosis determined in two measurements, histological quantification of total area of collagen deposition through Picro Sirius Red staining and α-SMA staining (Fig 2B–2D, 2G and 2H). Consistent with the reduction of collagen protein, livers from animals treated with anti-RSPO3 antibody showed a significant decrease in collagen I (61% p<0.001) and collagen III (53%, p<0.001) mRNAs (Fig 2E and 2F). When staining for β-catenin in livers from animals treated with different anti-RSPO antibodies, we found that only treatment with OMP-131R10 showed an inhibition of β-catenin cytoplasmic and nuclear translocation (Fig 2I). Little difference from either anti-RSPO1 or 2 antibody treatment was observed in comparison with control IgG (Fig 2B–2D and 2G–2I). These results establish RSPO3 as an important pro-fibrotic protein and provide a functional role for RSPO3 in liver fibrosis. The results also support that the anti-fibrotic activity of anti-RSPO3 is associated with inhibition of Wnt/β-catenin signaling.

### OMP-131R10 is a therapeutic agent for liver, lung, and skin fibrosis

**Fig 3:**
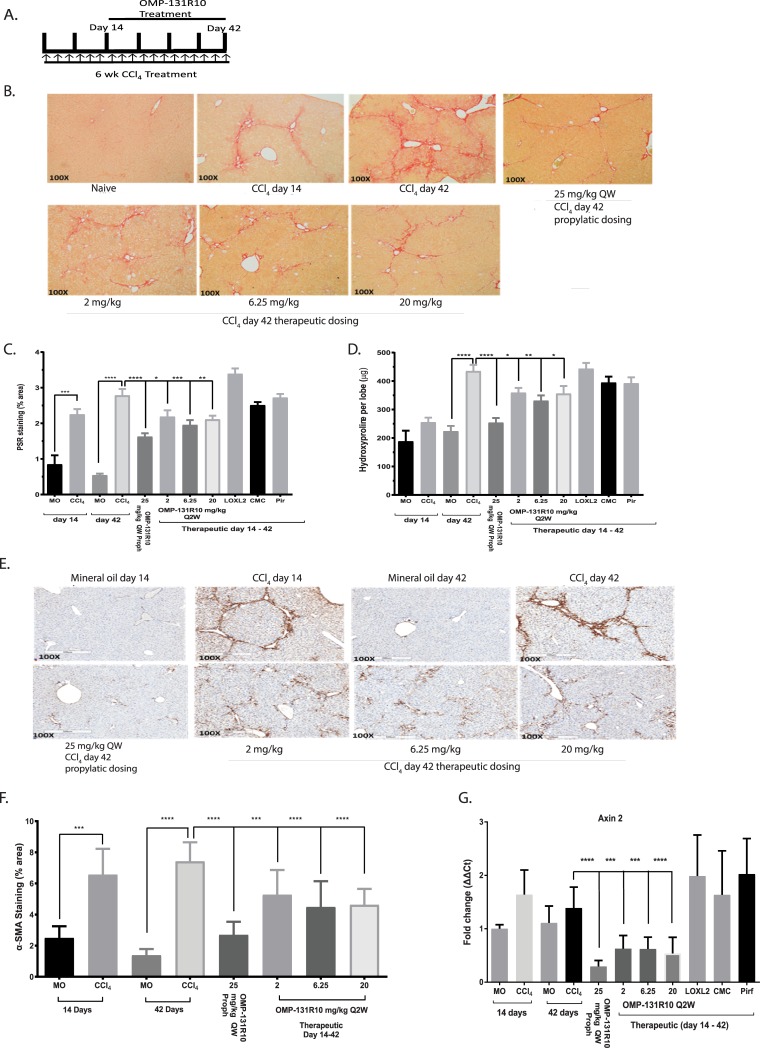
Anti-RSPO3 OMP-131R10 is a therapeutic agent for liver fibrosis. **A,** Treatment scheme of OMP-131R10 in CCl_4_ liver fibrosis model. **B,** Picro Sirius Red staining of normal and CCl_4_ induced mouse livers treated with OMP-131R10. **C**, Quantification of Picro Sirius Red staining area and liver collagen content. **D,** Quantification of liver collagen content. **E,** Immunohistochemistry analysis of α-SMA in the livers of normal and CCl_4_ induced mouse livers treated with OMP-131R10. **F,** Quantification of α-SMA staining area. **G,** RT-PCR of Axin2. Liver fibrosis was induced by CCl_4_ injection three times weekly for 6 weeks. The mice were treated from day 1 with OMP-131R10 at 25 mg/kg once a week (n = 10 animals per group except mineral oil control n = 4). Liver fibrosis was induced by CCl_4_ injection three times weekly for 6 weeks. For prophylactic treatment mice were treated from Day 1 with OMP-131R10 at 25mg/kg once a week (n = 10 animals per group except mineral oil control n = 7). For therapeutic treatment mice were treated from day 14 with OMP-131R10 at 2, 6.25, and 20 mg/kg once every two weeks. At week 6, the study was terminated. Data is shown as mean ± SEM. Statistical comparison was done using one-way ANOVA with Dunnett’s multiple comparisons test using the Isotype as a control. * = p < 0.05, ** = p < 0.01, **** = p < 0.0001.

After having established a functional role for RSPO3 in liver fibrosis, we next sought to examine if OMP-131R10 could be a therapeutic agent to treat fibrosis and whether OMP-131R10 possesses broad anti-fibrotic activity. Thus we tested OMP-131R10 in the models of CCl_4_-induced liver fibrosis and bleomycin-induced lung fibrosis with a therapeutic (dosed at 2, 6.25, and 20 mg/kg once every two weeks initiated 14 days after CCl_4_ or 7 days after bleomycin) rather than a prophylactic treatment paradigm. In addition, an anti-LOXL2 antibody, the same antibody was used in clinical trials for NASH, and pirfenidone, an approved drug for IPF, were included in the study as controls [22, 23]. For the CCl_4_ study, we also included a 25 mg/kg once a week prophylactic arm, the same treatment as used in Fig 2, to control for the variability between studies (Fig 3A). All three therapeutic doses (2, 6.25, and 20 mg/kg) of OMP-131R10 demonstrated significant inhibition of fibrosis, measured by histological collagen staining, hydroxyproline quantification, and α-SMA staining (Fig 3B–3F), while little anti-fibrotic activity was observed from either pirfenidone or anti-LOXL-2 antibody treatment (Fig 3B–3F). Prophylactic dosing of OMP-131R10 at 25mg/kg once a week provided almost identical protection as observed in Fig 2B and 2C, suggesting good consistency between two studies. The lack of dose response from OMP-131R10 suggests that the optimal therapeutic dose is likely equal to or lower than 2 mg/kg (Fig 3C and 3D). Consistent with the anti-fibrotic effect, inhibition of Wnt target gene Axin2 expression in livers from treated animals was maximal at all three doses of OMP-131R10 (Fig 3G), supporting maximal target inhibition at the lowest dose. Neither anti-LOXL2 antibody nor pirfenidone seemed to affect Axin2 expression (Fig 3G). These results together with findings shown in Figs 1 and 2 support that anti-fibrosis activity of OMP-131R10 is mediated by the inhibition of Wnt/β-catenin signaling.

**Fig 4:**
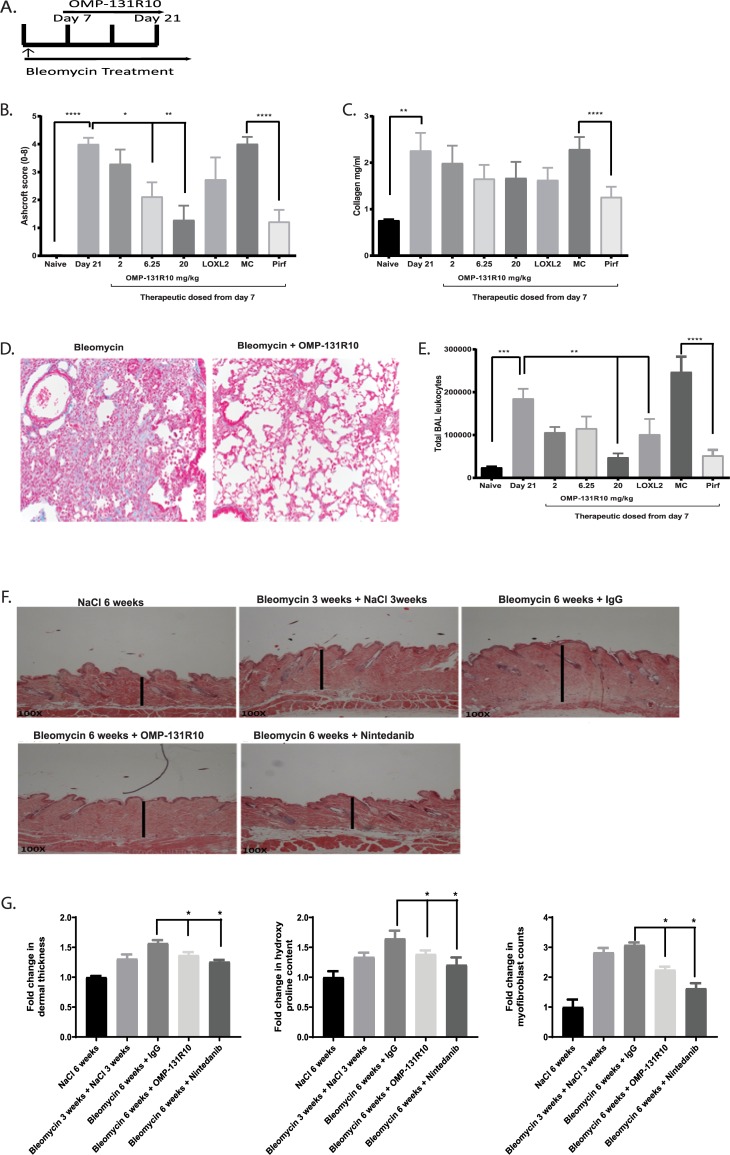
Anti-RSPO3 OMP-131R10 is a therapeutic agent for lung and skin fibrosis. **A,** Treatment scheme of OMP-131R10 in bleomycin-induced lung fibrosis model. **B,** Quantification of fibrosis using Ashcroft score. **C,** Quantification of lung collagen content. **D,** Representative Trichrome Staining on mouse lungs. **E,** Quantification of BAL leukocytes. **F,** Dermal thickness histology. **G,** Quantification of dermal thickness, myofibroblast content and hydroxyproline content. Skin fibrosis was induced by daily intradermal injections of bleomycin for 6 weeks. The mice were treated from day 14 with anti-RSPO3 (OMP-131R10) antibodies at 25 mg/kg once a week (n = 8 animals per group). At week 6, study was terminated and dermal thickness measured via histological methods data is shown as fold change in dermal thickness compared to the naïve. Data is shown as mean ± SEM. Statistical comparison was done using paired student t-tests. * p < 0.05.

We next asked if OMP-131R10 could affect fibrosis in other organs. As we observed in CCl_4_ livers, RSPO3 was found to be highly elevated in bleomycin induced lung and skin fibrosis (S8 and S9 Figs). We tested that in the bleomycin-induced lung fibrosis model with the same doses [24] (Fig 4A). We observed that OMP-131R10 dose-dependently inhibited fibrosis in lung as determined by Ashcroft scoring, a histological quantification of lung fibrosis, and trichrome staining (Fig 4B and 4D). At the highest dose tested, 20mg/kg Q2W, OMP-131R10 demonstrated a strong anti-fibrotic effect, similar to the effect of a clinically relevant dose of pirfenidone (Fig 4B). Although anti-LOLX2 was reported to have anti-fibrotic activity in a similar model, no statistically significant anti-fibrosis activity was seen in this study (Fig 4B). In collagen content analysis, a trend towards fibrosis inhibition by OMP-131R10 was observed, but statistical significance was not reached (Fig 4C). Inflammation in response to injury is believed to be an important mechanism in the development of fibrosis. We assessed the effect of OMP-131R10 on inflammatory response. Treatment with OMP-131R10 significantly decreased the numbers of total bronchoalveolar lavage (BAL) leukocytes (Fig 4E). At 20 mg/kg Q2W, the dose at which the strongest anti-fibrosis was observed, OMP-131R10’s effect was highly comparable with pirfenidone treatment (Fig 4E).

Accumulating evidence suggest that fibrosis across organs often share core common mechanisms. We next further extended our study to bleomycin-induced skin fibrosis model [25]. OMP-131R10 was found to significantly inhibit all three fibrotic readouts including skin thickness, collagen deposition, and number of α-SMA positive cells (Fig 4F and 4G). Together these data confirm a broad and critical role for RSPO3 in fibrosis.

### RSPO3 is highly elevated in both IPF lungs and livers of NASH patients with advanced fibrosis

**Fig 5:**
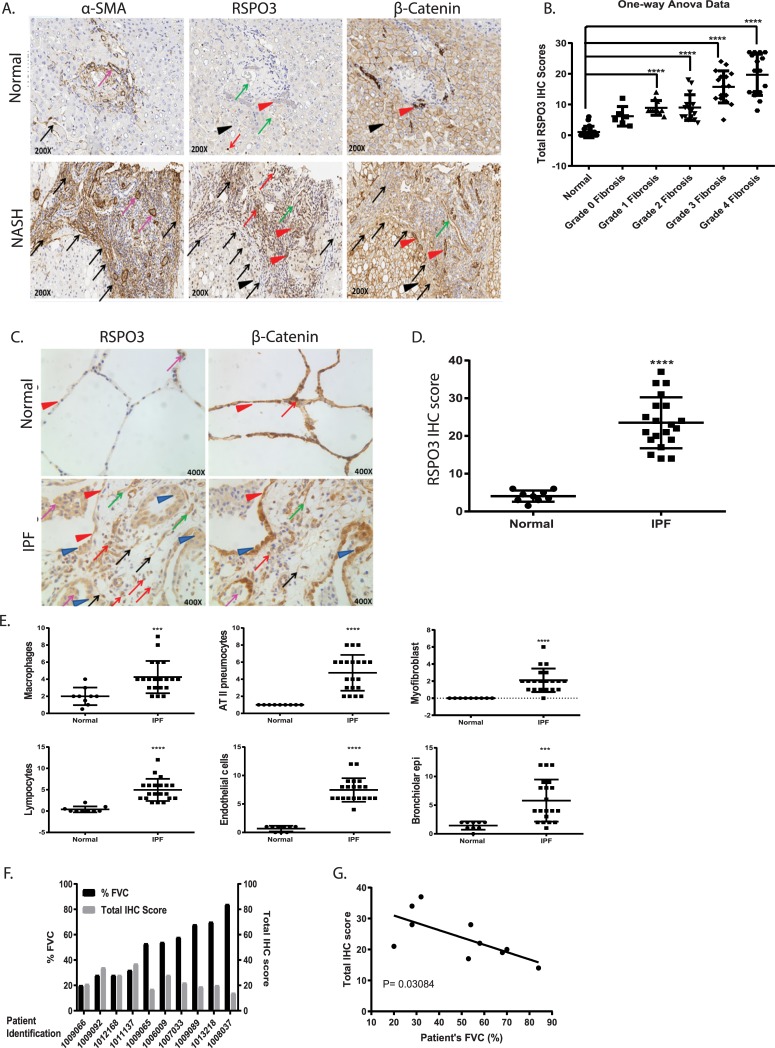
RSPO3 is highly elevated in both IPF lungs and livers of NASH patients with advanced fibrosis. **A**, Representative immunohistochemistry of α-SMA, RSPO3 and β-catenin in human normal and NASH liver. Smooth muscle cells (purple arrows); Hepatic stellate cells/myofibroblasts (black arrows); Inflammatory cells (red arrows); Bile ductular cells (red arrowhead); Blood vessel and sinusoidal endothelial cells (green arrows); Hepatocytes (black arrowhead). **B,** Quantification of RSPO3 IHC scoring in NASH livers with different grades of fibrosis (6–18 samples per group). **C,** Immunohistochemistry of RSPO3 and β-catenin in human normal and IPF lung. Macrophages (purple arrows); AT II (blue arrowhead); Myofibroblasts (black arrows); Lymphocytes (red arrows); Endothelial cells (green arrows); Bronchiolar epithelial cells (red arrowhead). **D,** Quantification of RSPO3 IHC scoring in normal (n = 9) and IPF lung (n = 21). **E,** Quantification of RSPO3 expression by IHC in different IPF lung cell types. **F and G,** Correlation of RSPO3 IHC score and lung function. Data is shown as mean ± SEM. Statistical comparison was done using one-way ANOVA with Bartlett's test using the grade 0 as a control. ****P <0.0001.

After we showed that RSPO3 is highly expressed in fibrotic lesions and plays an important role in the development of fibrosis in animal models of fibrosis for multiple organs, we then wanted to determine the expression of RSPO3 in human fibrotic tissues. It is reasonable to suggest that upregulation of RSPO3 expression in human fibrotic tissues could have functional implications. We therefore performed IHC staining for RSPO3 in fibrotic tissues from patients. For liver fibrosis, we analyzed liver samples from NASH patients with different stages of fibrosis, F0-F4. As shown in Fig 5A, RSPO3 expression was elevated in NASH livers, particularly within fibrotic lesions as indicated by staining for α-SMA. Quantification analysis showed that the highest RSPO3 expression was found in patients with advanced fibrotic stages, F3 and F4 (Fig 5B), suggesting a correlation of RSPO3 expression with fibrosis progression and severity. Strong staining signals seemed to come from hepatocytes, inflammatory cells and activated HSC (Fig 5A). The substantial elevation of RSPO3 in fibrosis stages F3 and F4 is in an agreement with what we found in CCl_4_ fibrotic livers, which features the bridging fibrosis pattern that resembles stages 3 fibrosis in human NASH patients.

Likewise, in a similar analysis we found that the overall expression of RSPO3 in IPF lungs was significantly higher than control subjects (Fig 5C and 5D). In control lungs, RSPO3 was mainly detected in macrophages and type II alveolar epithelial cells (Fig 5C). IPF lungs were characterized, among other histological features, by numerous fibroblastic foci in which myofibroblasts are in direct contact with damaged alveolar epithelial cells containing high number of hyperplastic alveolar type II cells. In addition to macrophages and hyperplastic ATII cells, high expression of RSPO3 was also found in subsets of lymphocytes and myofibroblasts in IPF lungs (Fig 5C and 5E). With patients whose lung function information is available, we found that the expression levels of RSPO3 were inversely correlated with their lung function (Fig 5F and 5G). These results suggest a potentially important role for RSPO3 in human liver and lung fibrosis.

### RSPO3 induces multiple fibrotic mediators

**Fig 6:**
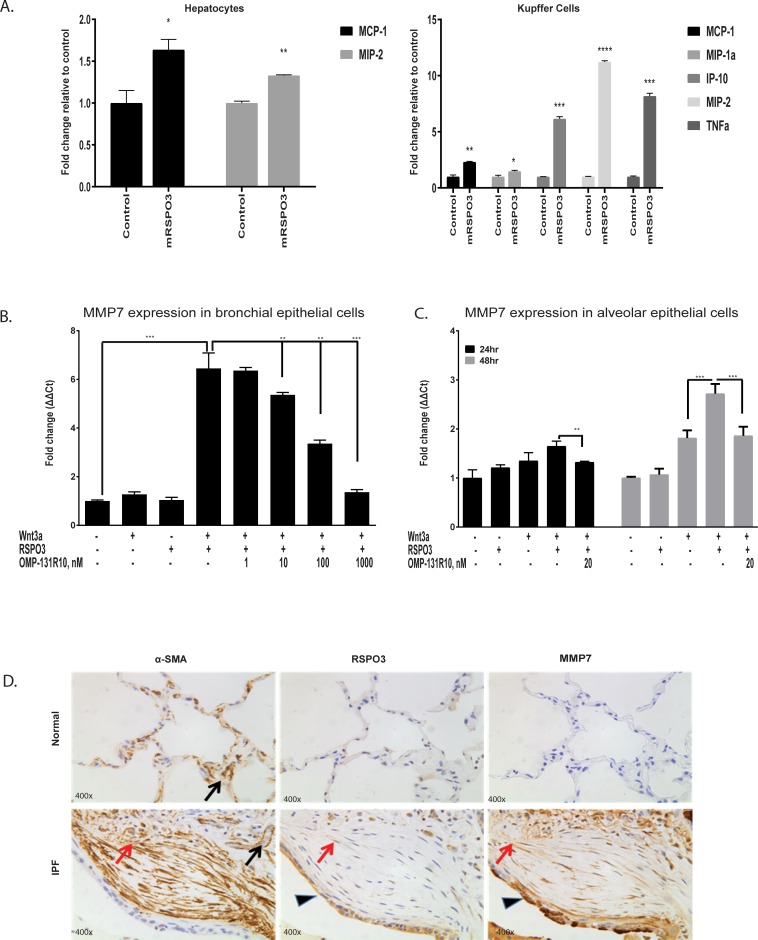
RSPO3 induces multiple fibrotic mediators. **A**, Cytokine secretion in mouse hepatocytes and Kupffer cells treated with RSPO3. **B,** RT-PCR data for MMP-7 in bronchial epithelial cells treated with RSPO3. **C**, RT-PCR data for MMP-7 in alveolar epithelial cells treated with RSPO3. **D,** Expression of α-SMA, RSPO3, and MMP-7 was analyzed by IHC in normal human and IPF patient lungs. In normal human lungs, α-SMA expression was observed in vascular smooth muscle (black arrow), RSPO3 or MMP-7 was weakly or rarely present in alveolar epithelium. In IPF patient lungs, α-SMA was present in vascular smooth muscle and myofibroblasts (red arrows) in fibroblastic foci. In addition, both RSPO3 and MMP-7 were highly expressed in hyperplastic type II epithelial cells (arrowhead). Data is shown as mean ± SEM. Statistical comparison was done using paired student t-tests. *p < 0.05, ** p < 0.01, *** p < 0.001, **** p < 0.0001.

The mechanisms of Wnt/β-catenin driven fibrosis seem to be complex and are not fully understood [26]. We showed the RSPO3 pro-fibrotic activity is strongly associated with the activation of Wnt/β-catenin signaling. To further understand how RSPO3 promotes fibrosis, we focused on cells that were shown to express high levels of RSPO3 under fibrotic conditions. Interestingly, these cells are also known to be involved in fibrosis. MCP-1 and MIP-2 were induced by RSPO3 in hepatocytes (Fig 6A). Additional chemokines and cytokine, IP-10, MIP-1a and TNFα, were found to be elevated by RSPO3 in Kupffer cells (Fig 6A). All of these chemokines and cytokine have been shown to play an important role in driving liver fibrosis [27–29]. However, no significant effect from RSPO3 was observed in mouse hepatic stellate cells.

MMP-7, a Wnt target gene, has been shown to play an important role in lung fibrosis [30]. Levels of MMP-7 are elevated in IPF lungs and in circulation of IPF patients [31, 32]. Serum MMP-7 concentration was found to be significantly correlated with disease severity and survival in IPF patients [33]. Here we show that MMP-7 is induced by RSPO3 in human primary bronchial and alveolar epithelial cells (Fig 6B and 6C). Through IHC analysis, we found that MMP-7 and RSPO3 were both significantly up-regulated in IPF lung, in particular in hyperplastic alveolar type II (ATII) epithelial cells (Fig 6D), suggesting that RSPO3 may play a regulatory role in MMP-7 expression in IPF.

## Discussion

Here we provide compelling evidence that RSPO3 plays a critical role in fibrosis of multiple organs, thereby defining a specific anti-fibrosis treatment strategy with an anti-RSPO3 antibody. Our understanding of the role of RSPO3 began when we tested three members of the RSPO family using specific monoclonal antibodies in well-established *in vivo* murine model of liver fibrosis. We demonstrated that only anti-RSPO3 antibody significantly ameliorated liver fibrosis through inhibition of the Wnt/β-catenin signaling, although all three RSPO proteins function primarily through the enhancement of the pathway. We extended our study to other organs and showed that OMP-131R10 was efficacious in inhibiting lung and skin fibrosis *in viv*o. The significant therapeutic effect of OMP-131R10 in fibrosis models from different organs suggest that RSPO3 functions at a core and common fibrotic mechanism that could be a promising target for therapy development. Our approach using monoclonal antibodies overcomes the challenges associated with genetic manipulation of RSPO genes due to the severe phenotypes caused by gene knockout. As cellular sources of RSPO proteins *in vivo* are poorly defined, in particular under diseased conditions, there is no clear strategy for conditional knockout approach.

Our findings that RSPO3 is highly elevated in human fibrotic lungs and livers, and that RSPO3 levels are significantly correlated with disease severity, provide supporting evidence that RSPO3 may play a role in lung and liver fibrosis in human. These findings seem to be more compelling when we consider what we have learned from our *in vivo* studies where the anti-fibrotic activity of anti-RSPO3 OMP131R10 was closely related to the overexpression of RSPO3 in the fibrotic lesions. Unlike what was previously reported where RSPO expression was measured in a Western blotting, we could not detect significant elevation of RSPO1 or RSPO2 in CCl_4_ livers by IHC [34]. The discrepancy could be due to different antibodies used and the sensitivity of different detection methods.

While the important role of Wnt/β-catenin signaling in the development of fibrosis has been well documented, the mechanism of how this pathway drives fibrosis is not completely clear. Increased proliferation and activation of mesenchymal cells such fibroblasts and hepatic stellate cells by the activation of Wnt/β-catenin signaling pathway were reported to contribute to the development of fibrosis [26]. In our *in vitro* study, we found little or no evidence that RSPO3 treatment affects any of potential pro-fibrotic activity in these cells. However, we found that multiple chemokines, MCP-1, MIP-1α, MIP-2, and cytokine TNFα were significantly induced by RSPO3 in hepatocytes or Kupffer cells. Both MCP-1 and MIP-1α were shown to drive liver fibrosis through increasing hepatic inflammation and activation of HSC [27–29]. MIP-2 from Kupffer cells recruits and activates neutrophil, and induces inflammation in response to liver injury [28]. Kupffer-derived TNFα was found to enhance HSC cell survival, which in turn promotes liver fibrosis [35]. MMP-7 induction by RSPO3 could be an important mechanism by which RSPO3 promotes lung fibrosis, which deserves further study. Given the strong correlation of MMP-7 level in circulation with IPF disease severity, we believe the co-expression of RSPO3 and MMP-7 in fibroblastic foci of IPF lung could potentially have important functional implication for RSPO3.

The importance of Wnt/β-catenin pathway in fibrosis and the potential of targeting this pathway for therapeutic development have been described and proposed [4, 36]. However, a clear targeting strategy remains to be defined. With many cellular components involved, the Wnt pathway is complex and yet tightly regulated [37]. As one of the major developmental pathways, Wnt/β-catenin signaling plays a regulatory role in adult tissue homeostasis in multiple organs. Therefore, safety concerns for non-selective and global inhibition of β-catenin activity could be an issue [38]. We and others have reported that RSPO3 is highly expressed in certain human tumors, and RSPO3 antagonism inhibits tumor growth in animal models [39,40]. In fact, OMP131R10 has been tested in a phase 1 human trial. Low incidence of GI adverse events was observed in cancer patients who were treated with relatively high doses of OMP-131R10. As for the bone effect, a few patients experienced changes in bone metabolism with the elevation of bone resorption marker (β-CTX) and decrease of bone formation marker (P1NP). These data suggest that RPSO3 is not a dominant player in regulating Wnt signaling in these normal tissues, which helps improve the safety profile for OMP-131R10.

The combination of *in vivo* efficacy study results, target expression data, and pathway activation analysis in human fibrotic tissues provides strong support for a future clinical trial for IPF, and possibly for NASH with advanced fibrosis or cirrhosis and systemic scleroderma with OMP-131R10. The encouraging safety and tolerability data obtained so far in the phase 1a oncology trial support considering the anti-RSPO3 strategy for additional indications.

## Supporting information

S1 Fig Validation of specificity of RSPO1, 2, 3 antibodies by WB. Specificity of RSPO1,2,3 antibodies (Atlas Antibody, HPA046154, HPA024764, HPA029957) was tested by Western Blot using recombinant human proteins (A) and HEK293T cells overexpressing human RSPO proteins (B).
(DOCX)

S2 Fig Validation of specificity of RSPO1, 2, 3 antibodies by IHC on cell pellets overexpressing human (h) or mouse (m) RSPO1, 2, 3 proteins. Specificity of RSPO1, 2, 3 antibodies, at varying titrations, was evaluated on HEK293T cell pellets transiently transfected with human (A, B, C) or mouse (D, E, F) RSPO1, 2, 3 plasmids. Control was HEK293T cells transiently transfected with empty vector (Origene, PS100001). Each isoform antibody specifically stained the cell pellets overexpressing its corresponding antigen only. Pictures were taken at 200x magnification.
(DOCX)

S3 Fig Validation of specificity of RSPO1, 2, 3 antibodies on human normal colon by IHC. Human normal colon was stained with RSPO1-3 antibodies (Atlas Antibody), at varying titrations, together with normal rabbit polyclonal IgG as isotype control. All of 3 isoform antibodies generated clear signal in villi epithelium (arrow). Endothelium (arrowhead) and lymphocytes (*) were also positively stained by RSPO3antibody. A titration of 1:50 for RSPO1 and 2, 1:250 for RSPO3 was chosen for the future staining. Pictures were taken at 100x magnification.
(DOCX)

S4 Fig Validation of specificity of RSPO1 antibody via antigen blocking. RSPO1 antibody was pre-incubated, overnight at 4 degree, with recombinant mouse or human RSPO1 protein (R&D systems, 3474-RS, 4645-RS/CF) at a molar ratio of 1:10 prior to IHC staining. Specific immunostaining of RSPO1 on mouse RSPO1 overexpressed HEK293T cells (A), normal (B) and CCl_4_ injured (C) mouse livers, human RSPO1 overexpressed HEK293T cells (D), epithelium (arrows) in normal (E) and IPF patient (F) lungs was efficiently blocked by recombinant RSPO1 proteins. Pictures were taken at 200x magnification.
(DOCX)

S5 Fig Validation of specificity of RSPO2 antibody via antigen blocking. RSPO2 antibody was pre-incubated, overnight at 4 degree, with recombinant mouse or human RSPO2 protein (R&D systems, 6946-RS/CF, 3266-RS/CF) at a molar ratio of 1:10 prior to IHC staining. Specific immunostaining of RSPO2 on mouse RSPO2 overexpressed HEK293T cells (A), normal (B) and CCl_4_ injured (C) mouse livers, human RSPO2 overexpressed HEK293T cells (D), epithelium (arrows) in normal human colon (E) and kidney (F) was efficiently blocked by recombinant RSPO2 proteins. Pictures were taken at 200x magnification.
(DOCX)

S6 Fig Validation of specificity of RSPO3 antibody via antigen blocking. RSPO3 antibody was pre-incubated, overnight at 4 degree, with recombinant mouse or human RSPO3 protein (R&D systems, 4120-RS/CF, 3500-RS/CF) at a molar ratio of 1:10 prior to IHC staining. Specific immunostaining of RSPO3 on mouse RSPO3 overexpressed HEK293T cells (A), normal (B) and CCl_4_ injured (C) mouse livers, human RSPO3 overexpressed HEK293T cells (D), epithelium (arrows) in normal human lung (E), and hyperplastic type II epithelial cells (arrow)/infiltrated lymphocytes (*)/ myofibroblasts (arrowhead) in IPF patient lung (F) was efficiently blocked by recombinant RSPO3 proteins. Pictures were taken at 200x magnification.
(DOCX)

S7 Fig R-Spondin Antibody Specificity. Anti-RSPO antibodies' inhibitory activity was examined in mouse bronchial epithelial cells treated with WNT3A supplemented with mouse RSPO1, RSPO2, or RSPO3 in the presence or absences of Anti-RSPO antibodies. Modulation of Wnt/RSPO target genes Axin2 was monitored by qPCR.
(DOCX)

S8 Fig RSPO1-3 expression in normal and bleomycin treated mouse lungs. In normal mouse lungs, moderate RSPO1-3 expression was mainly found in bronchiolar epithelium (red arrow) and alveolar macrophages (black arrow). In addition, weak RSPO2 & 3 in blood vascular endothelium (green arrow) and weak RSPO3 in pneumocytes (pink arrow) were also observed. In bleomycin-injured mouse lungs, up-regulated RSPO1-3 was observed in infiltrating inflammatory cells (red arrowhead) & lymphocytes (_*_), hypertrophic and hyperplastic type II pneumocytes (black arrowhead) and alveolar macrophages (black arrow).
(DOCX)

S9 Fig RSPO1-3 expression in normal & bleomycin-treated mouse skins. In both normal & bleomycin injured mouse skins, RSPO1-3 expression was mainly found in epidermis & hair follicle (black arrow), sebaceuos glands (red arrow), adipocytes (green arrow), and muscle (_*_). In addition, weak RSPO2 & 3 in blood vascular endothelium (pink arrow) was also observed.
(DOCX)

S1 Data (DOCX)
