# Supplementary material for: Targeting the Wnt signaling pathway through R-spondin 3 identifies an anti-fibrosis treatment strategy for multiple organs
Source: PLoS One. 2020 Mar 11;15(3):e0229445. doi: 10.1371/journal.pone.0229445 (PMC7065809; doi:10.1371/journal.pone.0229445)
Supplement: S1 Fig — Specificity of RSPO1,2,3 antibodies (Atlas Antibody, HPA046154, HPA024764, HPA029957) was tested by Western Blot using recombinant human proteins (A) and HEK293T cells overexpressing human RSPO proteins (B). (DOCX) [file pone.0229445.s001.docx]

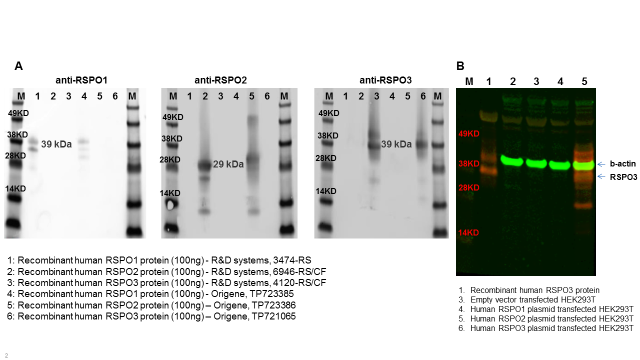
Figure S1. Validation of specificity of RSPO1, 2, 3 antibodies by WB.

Specificity of RSPO1,2,3 antibodies (Atlas Antibody, HPA046154, HPA024764, HPA029957) was tested by Western Blot using recombinant human proteins (A) and HEK293T cells overexpressing human RSPO proteins (B).
