# Supplementary material for: Targeting the Wnt signaling pathway through R-spondin 3 identifies an anti-fibrosis treatment strategy for multiple organs
Source: PLoS One. 2020 Mar 11;15(3):e0229445. doi: 10.1371/journal.pone.0229445 (PMC7065809; doi:10.1371/journal.pone.0229445)
Supplement: S2 Fig — Specificity of RSPO1, 2, 3 antibodies, at varying titrations, was evaluated on HEK293T cell pellets transiently transfected with human (A, B, C) or mouse (D, E, F) RSPO1, 2, 3 plasmids. Control was HEK293T cells transiently transfected with empty vector (Origene, PS100001). Each isoform antibody specifically stained the cell pellets overexpressing its corresponding antigen only. Pictures were taken at 200x magnification. (DOCX) [file pone.0229445.s002.docx]

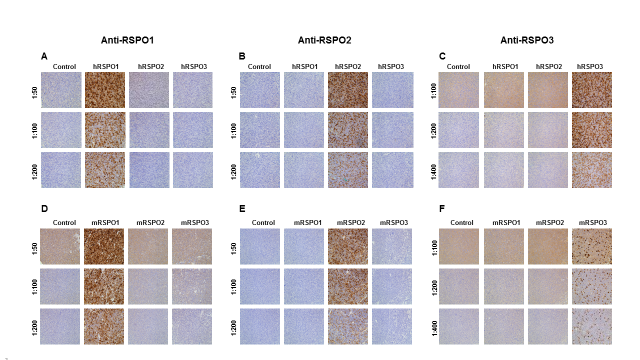


Figure S2. Validation of specificity of RSPO1, 2, 3 antibodies by IHC on cell pellets overexpressing human (h) or mouse (m) RSPO1, 2, 3 proteins.

Specificity of RSPO1, 2, 3 antibodies, at varying titrations, was evaluated on HEK293T cell pellets transiently transfected with human (A, B, C) or mouse (D, E, F) RSPO1, 2, 3 plasmids. Control was HEK293T cells transiently transfected with empty vector (Origene, PS100001). Each isoform antibody specifically stained the cell pellets overexpressing its corresponding antigen only. Pictures were taken at 200x magnification.
