# Supplementary material for: Targeting the Wnt signaling pathway through R-spondin 3 identifies an anti-fibrosis treatment strategy for multiple organs
Source: PLoS One. 2020 Mar 11;15(3):e0229445. doi: 10.1371/journal.pone.0229445 (PMC7065809; doi:10.1371/journal.pone.0229445)
Supplement: S3 Fig — Human normal colon was stained with RSPO1-3 antibodies (Atlas Antibody), at varying titrations, together with normal rabbit polyclonal IgG as isotype control. All of 3 isoform antibodies generated clear signal in villi epithelium (arrow). Endothelium (arrowhead) and lymphocytes (*) were also positively stained by RSPO3antibody. A titration of 1:50 for RSPO1 and 2, 1:250 for RSPO3 was chosen for the future staining. Pictures were taken at 100x magnification. (DOCX) [file pone.0229445.s003.docx]

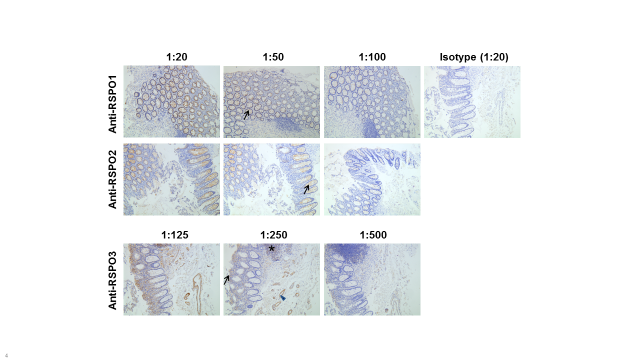


Figure S3. Validation of specificity of RSPO1, 2, 3 antibodies on human normal colon by IHC.

Human normal colon was stained with RSPO1-3 antibodies (Atlas Antibody), at varying titrations, together with normal rabbit polyclonal IgG as isotype control. All of 3 isoform antibodies generated clear signal in villi epithelium (arrow). Endothelium (arrowhead) and lymphocytes (*) were also positively stained by RSPO3antibody. A titration of 1:50 for RSPO1 and 2, 1:250 for RSPO3 was chosen for the future staining. Pictures were taken at 100x magnification.
