# Supplementary material for: Targeting the Wnt signaling pathway through R-spondin 3 identifies an anti-fibrosis treatment strategy for multiple organs
Source: PLoS One. 2020 Mar 11;15(3):e0229445. doi: 10.1371/journal.pone.0229445 (PMC7065809; doi:10.1371/journal.pone.0229445)
Supplement: S4 Fig — RSPO1 antibody was pre-incubated, overnight at 4 degree, with recombinant mouse or human RSPO1 protein (R&D systems, 3474-RS, 4645-RS/CF) at a molar ratio of 1:10 prior to IHC staining. Specific immunostaining of RSPO1 on mouse RSPO1 overexpressed HEK293T cells (A), normal (B) and CCl4 injured (C) mouse livers, human RSPO1 overexpressed HEK293T cells (D), epithelium (arrows) in normal (E) and IPF patient (F) lungs was efficiently blocked by recombinant RSPO1 proteins. Pictures were taken at 200x magnification. (DOCX) [file pone.0229445.s004.docx]

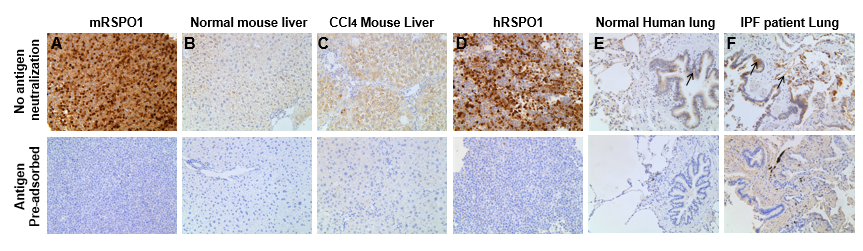


Figure S4. Validation of specificity of RSPO1 antibody via antigen blocking.

RSPO1 antibody was pre-incubated, overnight at 4 degree, with recombinant mouse or human RSPO1 protein (R&D systems, 3474-RS, 4645-RS/CF) at a molar ratio of 1:10 prior to IHC staining. Specific immunostaining of RSPO1 on mouse RSPO1 overexpressed HEK293T cells (A), normal (B) and CCl_4_ injured (C) mouse livers, human RSPO1 overexpressed HEK293T cells (D), epithelium (arrows) in normal (E) and IPF patient (F) lungs was efficiently blocked by recombinant RSPO1 proteins. Pictures were taken at 200x magnification.
