# Supplementary material for: Targeting the Wnt signaling pathway through R-spondin 3 identifies an anti-fibrosis treatment strategy for multiple organs
Source: PLoS One. 2020 Mar 11;15(3):e0229445. doi: 10.1371/journal.pone.0229445 (PMC7065809; doi:10.1371/journal.pone.0229445)
Supplement: S5 Fig — RSPO2 antibody was pre-incubated, overnight at 4 degree, with recombinant mouse or human RSPO2 protein (R&D systems, 6946-RS/CF, 3266-RS/CF) at a molar ratio of 1:10 prior to IHC staining. Specific immunostaining of RSPO2 on mouse RSPO2 overexpressed HEK293T cells (A), normal (B) and CCl4 injured (C) mouse livers, human RSPO2 overexpressed HEK293T cells (D), epithelium (arrows) in normal human colon (E) and kidney (F) was efficiently blocked by recombinant RSPO2 proteins. Pictures were taken at 200x magnification. (DOCX) [file pone.0229445.s005.docx]

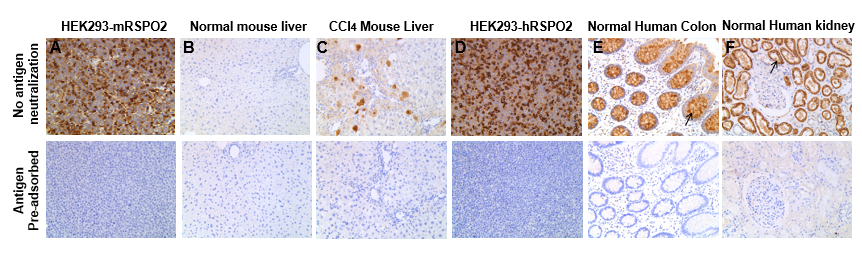
 Figure S5. Validation of specificity of RSPO2 antibody via antigen blocking.

RSPO2 antibody was pre-incubated, overnight at 4 degree, with recombinant mouse or human RSPO2 protein (R&D systems, 6946-RS/CF, 3266-RS/CF) at a molar ratio of 1:10 prior to IHC staining. Specific immunostaining of RSPO2 on mouse RSPO2 overexpressed HEK293T cells (A), normal (B) and CCl_4_ injured (C) mouse livers, human RSPO2 overexpressed HEK293T cells (D), epithelium (arrows) in normal human colon (E) and kidney (F) was efficiently blocked by recombinant RSPO2 proteins. Pictures were taken at 200x magnification.
