# Supplementary material for: Targeting the Wnt signaling pathway through R-spondin 3 identifies an anti-fibrosis treatment strategy for multiple organs
Source: PLoS One. 2020 Mar 11;15(3):e0229445. doi: 10.1371/journal.pone.0229445 (PMC7065809; doi:10.1371/journal.pone.0229445)
Supplement: S6 Fig — RSPO3 antibody was pre-incubated, overnight at 4 degree, with recombinant mouse or human RSPO3 protein (R&D systems, 4120-RS/CF, 3500-RS/CF) at a molar ratio of 1:10 prior to IHC staining. Specific immunostaining of RSPO3 on mouse RSPO3 overexpressed HEK293T cells (A), normal (B) and CCl4 injured (C) mouse livers, human RSPO3 overexpressed HEK293T cells (D), epithelium (arrows) in normal human lung (E), and hyperplastic type II epithelial cells (arrow)/infiltrated lymphocytes (*)/ myofibroblasts (arrowhead) in IPF patient lung (F) was efficiently blocked by recombinant RSPO3 proteins. Pictures were taken at 200x magnification. (DOCX) [file pone.0229445.s006.docx]

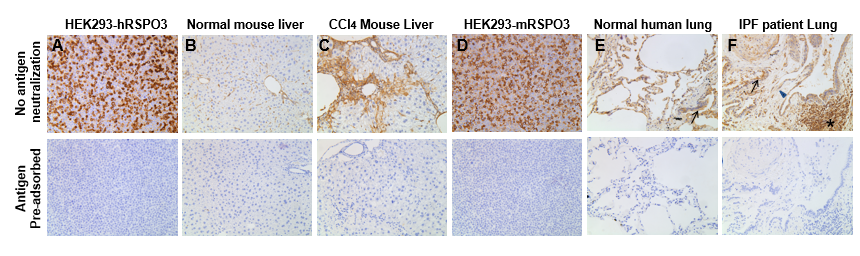


Figure S6. Validation of specificity of RSPO3 antibody via antigen blocking.

RSPO3 antibody was pre-incubated, overnight at 4 degree, with recombinant mouse or human RSPO3 protein (R&D systems, 4120-RS/CF, 3500-RS/CF) at a molar ratio of 1:10 prior to IHC staining. Specific immunostaining of RSPO3 on mouse RSPO3 overexpressed HEK293T cells (A), normal (B) and CCl_4_ injured (C) mouse livers, human RSPO3 overexpressed HEK293T cells (D), epithelium (arrows) in normal human lung (E), and hyperplastic type II epithelial cells (arrow)/infiltrated lymphocytes (*)/ myofibroblasts (arrowhead) in IPF patient lung (F) was efficiently blocked by recombinant RSPO3 proteins. Pictures were taken at 200x magnification.
