# Supplementary material for: Targeting the Wnt signaling pathway through R-spondin 3 identifies an anti-fibrosis treatment strategy for multiple organs
Source: PLoS One. 2020 Mar 11;15(3):e0229445. doi: 10.1371/journal.pone.0229445 (PMC7065809; doi:10.1371/journal.pone.0229445)
Supplement: S7 Fig — Anti-RSPO antibodies' inhibitory activity was examined in mouse bronchial epithelial cells treated with WNT3A supplemented with mouse RSPO1, RSPO2, or RSPO3 in the presence or absences of Anti-RSPO antibodies. Modulation of Wnt/RSPO target genes Axin2 was monitored by qPCR. (DOCX) [file pone.0229445.s007.docx]

Figure S7. R-Spondin Antibody Specificity

Anti-RSPO antibodies' inhibitory activity was examined in mouse bronchial epithelial cells treated with WNT3A supplemented with mouse RSPO1, RSPO2, or RSPO3 in the presence or absences of Anti-RSPO antibodies. Modulation of Wnt/RSPO target genes Axin2 was monitored by qPCR.
