# Supplementary material for: Targeting the Wnt signaling pathway through R-spondin 3 identifies an anti-fibrosis treatment strategy for multiple organs
Source: PLoS One. 2020 Mar 11;15(3):e0229445. doi: 10.1371/journal.pone.0229445 (PMC7065809; doi:10.1371/journal.pone.0229445)
Supplement: S8 Fig — In normal mouse lungs, moderate RSPO1-3 expression was mainly found in bronchiolar epithelium (red arrow) and alveolar macrophages (black arrow). In addition, weak RSPO2 & 3 in blood vascular endothelium (green arrow) and weak RSPO3 in pneumocytes (pink arrow) were also observed. In bleomycin-injured mouse lungs, up-regulated RSPO1-3 was observed in infiltrating inflammatory cells (red arrowhead) & lymphocytes (*), hypertrophic and hyperplastic type II pneumocytes (black arrowhead) and alveolar macrophages (black arrow). (DOCX) [file pone.0229445.s008.docx]

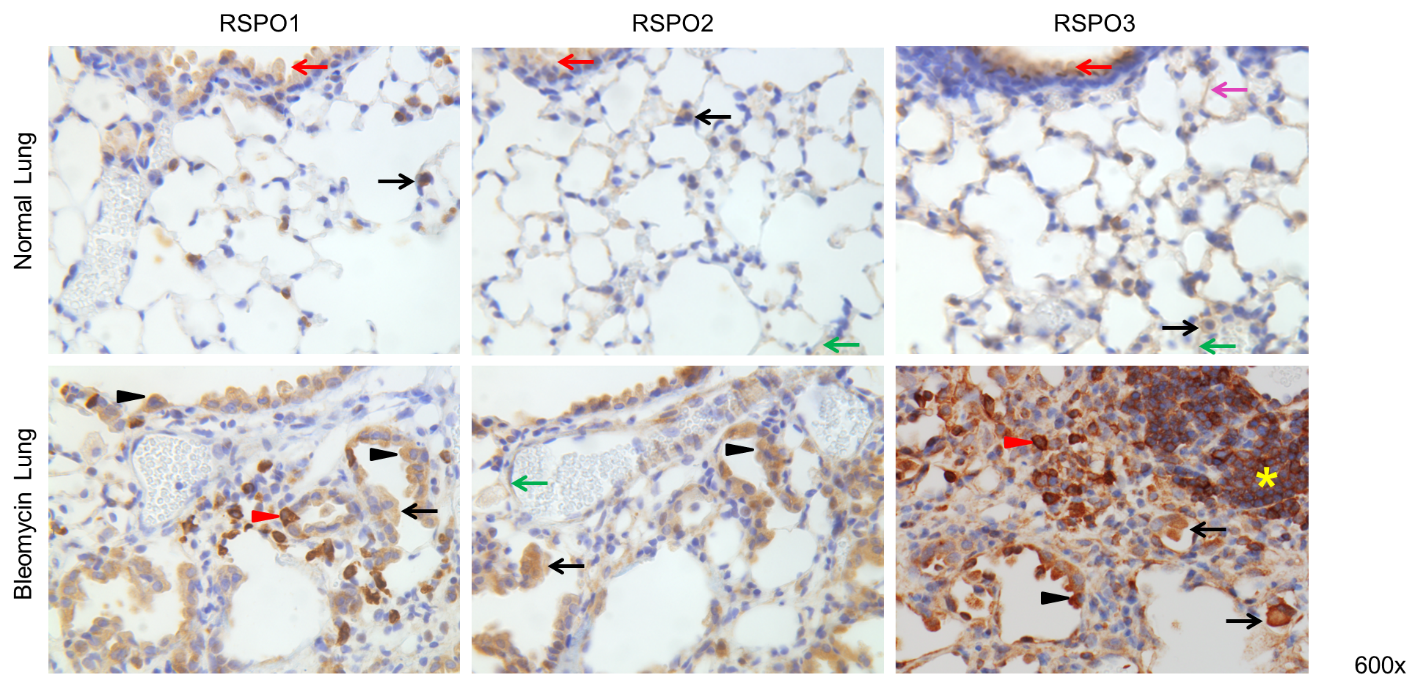


Figure S8. RSPO1-3 expression in normal and bleomycin treated mouse lungs.

In normal mouse lungs, moderate RSPO1-3 expression was mainly found in bronchiolar epithelium (red arrow) and alveolar macrophages (black arrow). In addition, weak RSPO2 & 3 in blood vascular endothelium (green arrow) and weak RSPO3 in pneumocytes (pink arrow) were also observed. In bleomycin-injured mouse lungs, up-regulated RSPO1-3 was observed in infiltrating inflammatory cells (red arrowhead) & lymphocytes (_*_), hypertrophic and hyperplastic type II pneumocytes (black arrowhead) and alveolar macrophages (black arrow).
