# Supplementary material for: Targeting the Wnt signaling pathway through R-spondin 3 identifies an anti-fibrosis treatment strategy for multiple organs
Source: PLoS One. 2020 Mar 11;15(3):e0229445. doi: 10.1371/journal.pone.0229445 (PMC7065809; doi:10.1371/journal.pone.0229445)
Supplement: S9 Fig — In both normal & bleomycin injured mouse skins, RSPO1-3 expression was mainly found in epidermis & hair follicle (black arrow), sebaceuos glands (red arrow), adipocytes (green arrow), and muscle (*). In addition, weak RSPO2 & 3 in blood vascular endothelium (pink arrow) was also observed. (DOCX) [file pone.0229445.s009.docx]

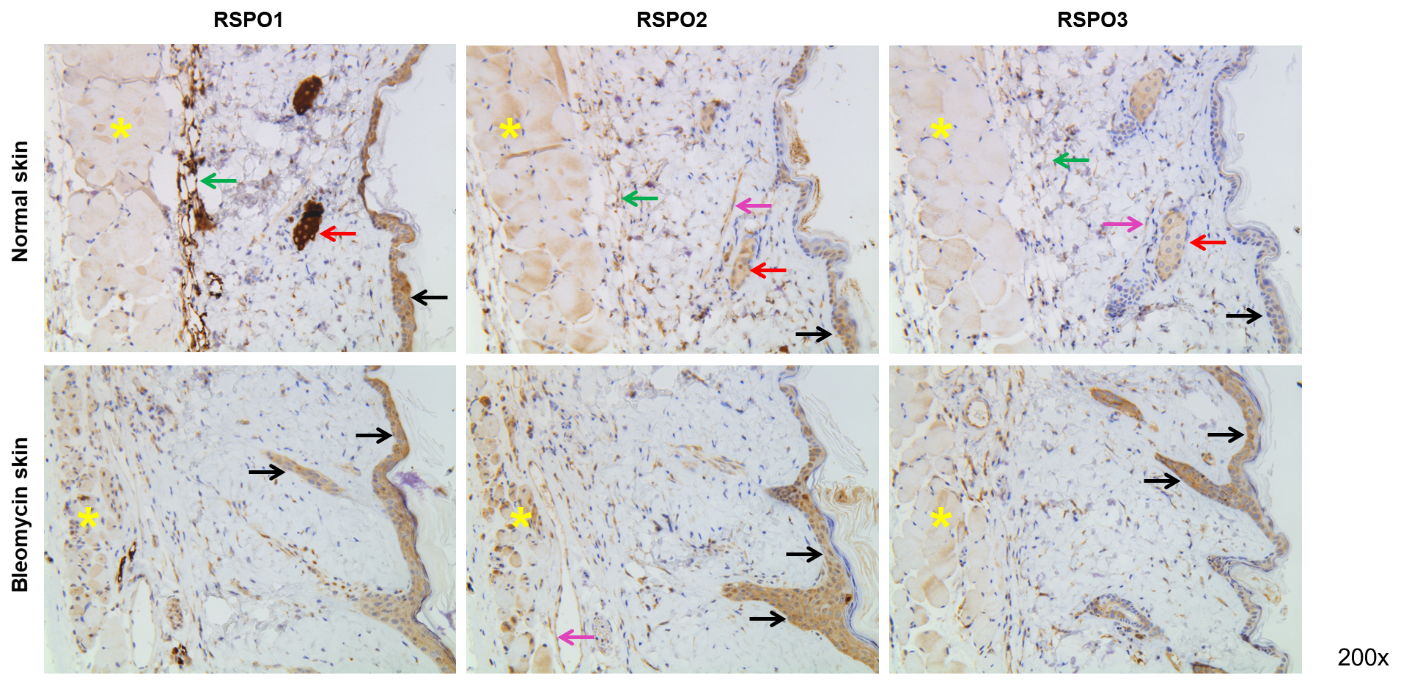


Figure S9. RSPO1-3 expression in normal & bleomycin-treated mouse skins.

In both normal & bleomycin injured mouse skins, RSPO1-3 expression was mainly found in epidermis & hair follicle (black arrow), sebaceuos glands (red arrow), adipocytes (green arrow), and muscle (_*_). In addition, weak RSPO2 & 3 in blood vascular endothelium (pink arrow) was also observed.
