# Supplementary material for: Targeting the Wnt signaling pathway through R-spondin 3 identifies an anti-fibrosis treatment strategy for multiple organs
Source: PLoS One. 2020 Mar 11;15(3):e0229445. doi: 10.1371/journal.pone.0229445 (PMC7065809; doi:10.1371/journal.pone.0229445)
Supplement: S1 Data — (DOCX) [file pone.0229445.s010.docx]

**Preparation of protein lysate and cell pellets from HEK293T cells transiently transfected with RSPO1-3 plasmids**

Human and Mouse RSPO1, 2, 3 plasmids (Origene, RC217921, MR226539, RC224177, MR216699, RC205016, and MR218621) were transiently transfected into HEK293T cells using lipofectamine 200 (Invitrogen, 11668019). 48-hr post transfection, cells were harvested by incubating with StemPro Accutase (Invitrogen# A1110501) for 5 min at 37°C, and number of cells/percentage of viability was measured using Couter Counter. For Western Blot, 10 million cells with viability of over 95% were lysed in lysis buffer containing one protease and phosphatase mini tablet (Pierce, 88669) and 10ml of NP40 cell lysis buffer (Invitrogen, FNN0021). Lysed proteins were separated on NuPAGE 4–12% Bis-Tris gel (Invitrogen, NP0321box). To prepare cell pellets for IHC, 20 million of viable transfected cells were spun down by centrifugation at 1200rpm for 5 minutes, and gently washed in PBS for 3 times. Cell pellets were fixed in 10% Neutral Buffered Formalin (NBF) at room temperature overnight, transferred to 70% ethanol next day for paraffin-embedding and processing.

**R-Spondin 3 Immunohistochemistry Scoring**

R-spondin 3 immunoreactivity in normal and IPF lungs was scored according to intensity (0 - 3) in alveolar macrophages, type II pneumocytes, myofibroblasts, lymphocytes, endothelial cells, and bronchiolar epithelial cells. Each cell population was scored from 0 - 4 to reflect relative quantity (0 = none, 1 = minimal, 2 = mild, 3 = moderate, and 4 = abundant). The score for each cell population was the product of intensity of staining and quantity of cells expressing the marker and ranged from 0 - 12. Final sample score was the sum of the scores of all cell populations examined in each sample and ranged from 0 - 60.

RSPO3 in normal and NASH livers was scored according to intensity (0-3) in bile duct epithelium, kupffer/stellate cells, lymphocytes, and endothelial cells. Each population was scored from 0-4 to reflect relative quantity in the sample and the score for each cell population was the product of intensity of staining and quantity of cells and ranges from 0-12. The total RSPO3 score was the sum of the scores of all cell populations examined and ranged from 0-60.
